# Kaposin-B Enhances the PROX1 mRNA Stability during Lymphatic Reprogramming of Vascular Endothelial Cells by Kaposi's Sarcoma Herpes Virus

**DOI:** 10.1371/journal.ppat.1001046

**Published:** 2010-08-12

**Authors:** Jaehyuk Yoo, Jinjoo Kang, Ha Neul Lee, Berenice Aguilar, Darren Kafka, Sunju Lee, Inho Choi, Juneyong Lee, Swapnika Ramu, Juergen Haas, Chester J. Koh, Young-Kwon Hong

**Affiliations:** 1 Departments of Surgery and Department of Biochemistry and Molecular Biology, Norris Comprehensive Cancer Center, Keck School of Medicine, University of Southern California, Los Angeles, California, United States of America; 2 Max-von-Pettenkofer Institut, Ludwig-Maximilians-Universität München, München, Germany; 3 Division of Pediatric Urology, Childrens Hospital Los Angeles and Keck School of Medicine, University of Southern California, Los Angeles, California, United States of America; University of California San Francisco, United States of America

## Abstract

Kaposi's sarcoma (KS) is the most common cancer among HIV-positive patients. Histogenetic origin of KS has long been elusive due to a mixed expression of both blood and lymphatic endothelial markers in KS tumor cells. However, we and others discovered that Kaposi's sarcoma herpes virus (KSHV) induces lymphatic reprogramming of blood vascular endothelial cells by upregulating PROX1, which functions as the master regulator for lymphatic endothelial differentiation. Here, we demonstrate that the KSHV latent gene kaposin-B enhances the PROX1 mRNA stability and plays an important role in KSHV-mediated PROX1 upregulation. We found that PROX1 mRNA contains a canonical AU-rich element (ARE) in its 3′-untranslated region that promotes PROX1 mRNA turnover and that kaposin-B stimulates cytoplasmic accumulation of the ARE-binding protein HuR through activation of the p38/MK2 pathway. Moreover, HuR binds to and stabilizes PROX1 mRNA through its ARE and is necessary for KSHV-mediated PROX1 mRNA stabilization. Together, our study demonstrates that kaposin-B plays a key role in PROX1 upregulation during lymphatic reprogramming of blood vascular endothelial cells by KSHV.

## Author Summary

Kaposi's sarcoma (KS) is the most common cancer in HIV-positive patients and KS-associated herpes virus (KSHV) was identified as its causing agent. We and others have discovered that when the virus infects endothelial cells of blood vessels, KSHV reprograms the cell type resembling endothelial cells in lymphatic vessels. Although endothelial cells of the blood vascular system and of the lymphatic system share functional similarities, the cell type-reprogramming does not occur under a normal physiological condition. Therefore, cell-fate reprogramming by the cancer-causing virus KSHV provides an important insight into the molecular mechanism for viral pathogenesis. Our current study investigates the molecular mechanism underlying the KSHV-mediated cell fate reprogramming. We identified that a KSHV latent gene kaposin-B plays an important role in KSHV-mediated regulation of PROX1 to promote PROX1 mRNA stability. This study will provide a better understanding on the tumorigenesis and pathogenesis of KS with a potential implication toward new KS therapy.

## Introduction

Kaposi's sarcoma (KS) is causally associated with human herpes virus (HHV)-8, also called KS-associated herpes virus (KSHV) [1]. KSHV develops various-sized KS tumors that are structurally accompanied by aberrant angiogenesis of slit-like vessels frequently containing red blood cells and inflammatory cells [2], [3]. KS tumor cells characteristically appear spindle-shaped and are believed to be derived from endothelial cells. KS tumor cells were initially proposed to originate from blood vascular endothelial cell (BEC) because of their expression of BEC-specific antigens [4], [5], [6], [7], [8], [9], [10]. Later, however, KS tumor cells were also found to express lymphatic endothelial cell (LEC)-specific markers such as VEGF receptor-3 (VEGFR-3/flt4) and podoplanin [11], [12], [13], [14], [15], [16], [17], [18], [19], arguing for their lymphatic origin. Recently, we and others have demonstrated that KSHV reprograms the transcriptional profile of BECs to resemble LECs by upregulation of PROX1, the master regulator for the LEC-differentiation [20], [21], [22], [23].

PROX1, the mammalian homolog of the *Drosophila* neuronal cell fate regulator Prospero, is a homeodomain transcription factor essential for development of a variety of organs, including the lymphatic system [24], [25], the liver [26], the lens [27], [28], the brain [29], [30], [31], [32], the ear [33], [34], [35], [36] and the heart [37], [38]. During early lymphatic development, endothelial cells in the cardinal vein exhibit a mixed phenotype of both BECs and LECs. A subset of venous endothelial cells begins to express PROX1 and migrates out to form the initial lymphatic vessels [24], [25]. This lymphatic differentiation process is found to be arrested in PROX1 knockout mice, which fail to develop the lymphatic system [24], [25]. We and others found that ectopic expression of PROX1 induces lymphatic reprogramming of post-developmental BECs [39], [40]. Therefore, PROX1 is thought to override the BEC phenotype by repressing BEC-specific markers and to induce lymphatic phenotypes by upregulating LEC-specific genes, functioning as the master control regulator for LEC differentiation.

Controlling mRNA stability is an important post-transcriptional regulatory process, which allows a rapid adjustment of the copy number of mRNAs by involving a sequence element called AU-rich element (ARE) [41], [42], [43], [44]. AREs are usually 50–150 nucleotide long and locate in the 3′-untranslated region (UTR) of mRNAs with a short half life, serving as an mRNA-destabilizing determinant by promoting degradation of mRNAs. Notably, ARE-containing mRNAs are found to represent as much as ∼8% of total mRNAs encoded in human cells and are involved in many essential biological processes such as signal transduction, cell growth and differentiation, immune responses, hematopoiesis and apoptosis [43], [44]. AREs are grouped into three classes based on the number and distribution of the core AUUUA pentamers [43], [45], [46]. Class I ARE genes contain several dispersed copies of the AUUUA motif within the AU-rich region and include c-myc, c-fos, cyclins A, B1 and D1 and interferon-γ. Class II ARE genes have at least 2 overlapping UUAUUUA(U/A)(U/A) motifs and include tumor necrosis factor (TNF)-α, interleukin (IL)-1β, IL-2, IL-3, granulocyte/macrophage colony-stimulating factor (GM-CSF), Cox-2 and VEGF. Finally, less characterized class III AREs do not contain the canonical AUUUA motif and are found in genes such as c-jun, GLUT, p53 and hsp70. Interestingly, while many cytokine-encoding mRNAs harbor the class II AREs, mRNAs encoding cell cycle regulators and transcription factors contain the class I and occasionally class III AREs [43]. Several ARE-binding proteins have been reported to either destabilize or stabilize ARE-containing mRNAs [43], [47]. Notably, HuR, embryonic lethal abnormal vision (ELAV)-like RNA-binding protein, is one of the best characterized ARE-binding proteins and stabilizes labile ARE-containing mRNAs such as c-fos, MyoD, p21, cyclins A, B1 and D1, TNF-α, GM-CSF and VEGF [42], [43], [48], [49], [50], [51], [52], [53], [54]. Predominantly present in the nuclei, HuR shuttles between the nucleus and cytoplasm in response to various internal and external stimuli, and its mRNA-stabilizing function has been attributed to its cytoplasmic localization [46], [50], [52], [55], [56].

Importantly, the KSHV latent gene kaposin-B has been shown to activate the p38/MK2 pathway and to stabilize various cytokine mRNA containing AREs [57], [58]. Kaposin-B can directly bind to MK2 and promote its kinase activity through its DR2 repeats and, in response to lipopolysaccharide (LPS), kaposin-B and MK2 were shown to be exported to cytoplasm [57], [58]. Kaposin-B and MK2/p38 proteins have been shown to enhance the stability of ARE-containing mRNAs such as GM-CSF and IL-6, leading to an enhanced production of cytokines and signaling proteins [57], [58]. However, the molecular mechanism underlying the kaposin-B/MK2-mediated stabilization of the ARE-containing mRNA remains to be better defined.

While study of endothelial cell fate reprogramming by KSHV has provided important insights into KS oncogenesis, the molecular mechanism underlying KSHV-mediated PROX1-upregulation has only begun to be elucidated. An interesting recent report has shown that Akt activation through gp130 receptor may play an important role in KSHV-induced lymphatic reprogramming [59]. Here, we found that PROX1 harbors an unusually long 3′-UTR that contains the canonical ARE, which functions as a PROX1 mRNA-destabilizing determinant. Moreover, we discovered that HuR protein physically binds and stabilizes PROX1 mRNA and that cytoplasmic localization of HuR protein is activated by kaposin-B. Together, our data demonstrate that kaposin-B plays a key role in KSHV-mediated PROX1 upregulation.

## Results

### KSHV infection is required for PROX1 upregulation in vascular endothelial cells

**Figure 1:**
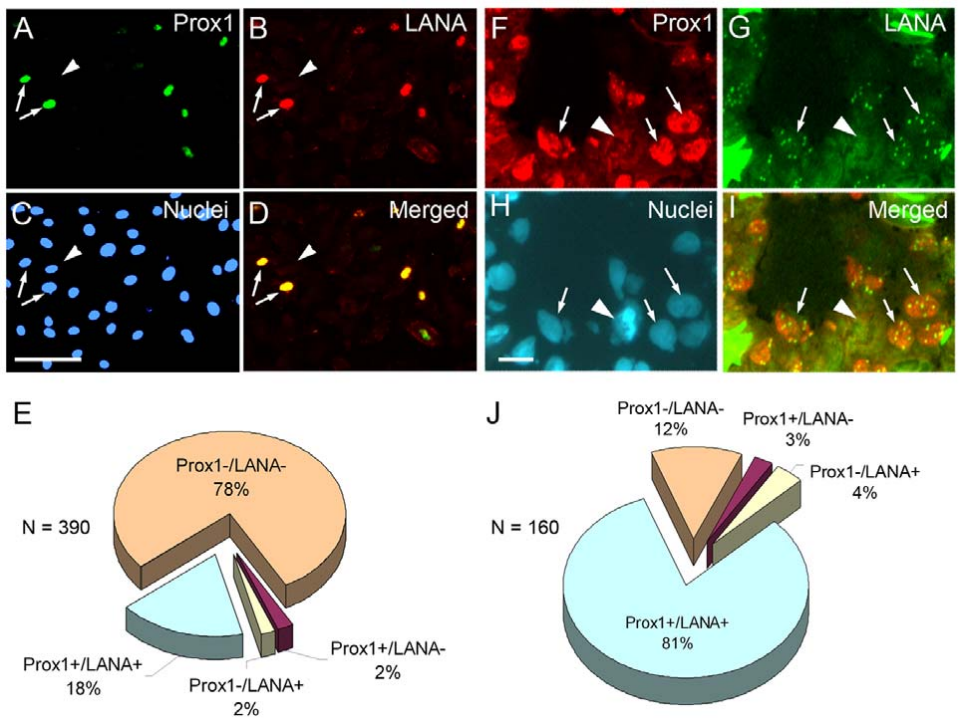
PROX1 is upregulated only in KSHV-infected endothelial cells *in vitro* and *in vivo*. (A-E) Cultured human dermal BECs were infected with KSHV for 7 days and subjected to immunofluorescent analyses against PROX1 (A), KSHV LANA/ORF73 (B) and nuclei (C). Merged image (D) shows that PROX1 expression is mainly detected in LANA-positive, KSHV-infected cells (arrows), but not in neighboring LANA-negative, uninfected cells (arrowhead). Bar, 100 µm. (E) Percent expression of PROX1 and/or LANA was analyzed in total 390 cells infected or not with KSHV. (F-I) Human cutaneous KS tumor section was immuno-stained against PROX1 (F), LANA (G) and nuclei (H). Merged image (I) shows that PROX1 expression is mainly detected in LANA-positive (speckled nuclear staining), KSHV-infected cells in KS tumor (arrows), but not in a neighboring LANA-negative uninfected cell (arrowhead). Percent expression of PROX1 and/or LANA was analyzed for total 160 nuclei in KS tumors and charted to evaluate the extent of co-expression of the two genes (J). Bar, 25 µm.

We and others have previously demonstrated that KSHV induces lymphatic reprogramming of vascular endothelial cells by upregulating PROX1 and that this PROX1-upregulation occurs in KSHV-infected cells *in vitro* [20], [21], [22], [23]. In this study, we further investigated the correlation between KSHV infection and PROX1 upregulation both *in vitro* and *in vivo*. For the *in vitro* study, we infected cultured human dermal BECs with KSHV for 7 days and performed immunofluorescent studies for PROX1 and LANA/ORF73, a KSHV viral protein that marks a latent KSHV-infection. We found that PROX1 was upregulated predominantly in LANA-positive, KSHV-infected BECs, but not in LANA-negative, uninfected neighboring BECs (Figure 1A-D). Our study revealed that ∼78% of the cells (n = 390) was double negative for PROX1 and LANA, and ∼18% double positive (Figure 1E), strongly correlating PROX1 upregulation with *de novo* KSHV-infection.

We next stained KS biopsy sections with anti-PROX1 and LANA antibodies to analyze co-expression of PROX1 and LANA in KS tumor cells (Figure 1F-I). We found that the majority of cells in KS tumors were infected with KSHV based on the characteristic LANA-speckles in KSHV-infected nuclei *in vivo* (Figure 1G) and that most of the LANA-positive, KSHV-infected cells upregulated PROX1. Out of 160 cells, ∼81% of the cells was double positive for PROX1 and LANA, ∼12% double negative, and only 3∼4% cells single-positive (Figure 1J), a finding consistent with the *in vitro* data. Together, our studies demonstrate that PROX1 upregulation occurs only in KSHV-infected cells.

### The KSHV latent gene kaposin-B induces PROX1-upregulation in LECs

**Figure 2:**
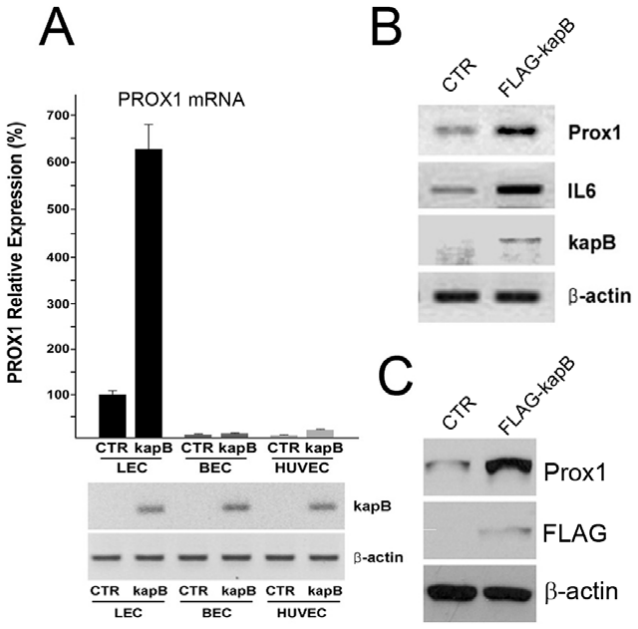
Kaposin-B upregulates PROX1 in primary lymphatic endothelial cells. (A) Regulation of PROX1 expression by kaposin-B in LECs, BECs and HUVECs. A control (CTR) or a kaposin B-expressing vector (kapB) was transfected into LECs, BECs and HUVECs for 16 hours and PROX1 mRNA level was determined and normalized against the internal control β-actin by using qRT-PCR analyses. Expression of kaposin-B and β-actin was also shown by semi-quantitative conventional RT-PCR. (B) A control (CTR) or a Flag-tagged kaposin-B (FLAG-kapB) vector was transfected into LECs for 48-hours and the expression of PROX1, IL6, kaposin-B and β-actin was determined by semi-quantitative RT-PCR analyses. (C) In a separate experiment, a control (CTR) or a Flag-tagged kaposin-B (FLAG-kapB) vector was transfected into LECs for 48-hours and protein expression of PROX1 and Flag-kaposin-B was determined by western analyses by using anti-PROX1, FLAG and β-actin antibodies.

We next investigated how KSHV induces PROX1-upregulation in endothelial cells. Since KSHV upregulates PROX1 in the latent stage, when only a few viral genes are known to be expressed, we hypothesized that one or more KSHV latent genes may be responsible for the activation of PROX1 expression and thus tested their ability to upregulate PROX1 in various endothelial cell backgrounds such as LECs, BECs and human umbilical venous endothelial cells (HUVECs). Real time RT-PCR (qRT-PCR) analyses revealed that ectopic expression of the KSHV latent gene kaposin-B in LECs significantly upregulated PROX1 (6∼7-fold) (Figure 2A). In comparison, kaposin-B did not notably induce PROX1 expression in either BECs or HUVECs (Figure 2A). From the same set of experiment, we investigated the effect of kaposin-B in the regulation of other lymphatic genes (podoplanin, VEGFR-3, LYVE-1, FGFR-3, SLC and p57) in LECs, BECs and HUVECs (Figure S1). Interestingly, we found that although kaposin-B alone did not seem to induce the lymphatic reprogramming as extensively as KSHV [20], [21], [22], [23], kaposin-B alone was able to partially modulate the expression of other LEC-signature genes in KSHV-infected endothelial cells. We next overexpressed kaposin-B in LECs and performed the semi-quantitative RT-PCR analyses against PROX1 and IL-6, a known kaposin-B target gene [57]. Like IL-6, PROX1 was also significantly upregulated by kaposin-B in LECs (Figure 2B). In a separate experiment, a Flag-tagged kaposin-B was transfected into LECs and the steady-state level of PROX1 protein was determined by western blot analyses (Figure 2C). Together, these data demonstrate that the KSHV viral gene kaposin-B can upregulate PROX1 expression in LECs, but not in BECs and HUVECs where the lymphatic-specific PROX1 is not expressed.

### PROX1 mRNA has an unusually long 3′ untranslated region

**Figure 3:**
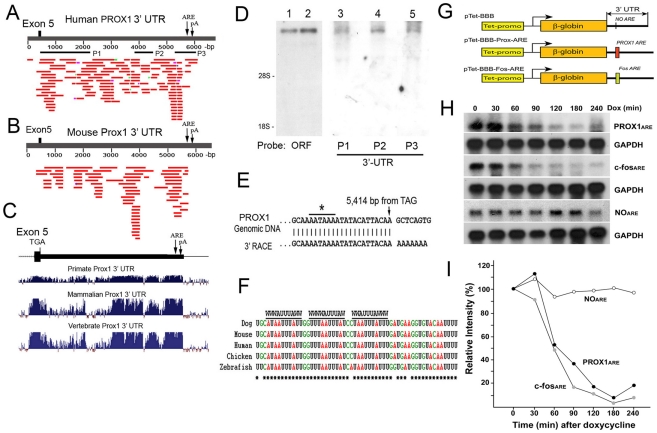
PROX1 mRNA has an unusually long 3′-untranslated region with a functional ARE that decreases its mRNA stability. (A) Alignment of 7-kb downstream genomic sequences of human PROX1 gene to human EST database identified numerous EST fragments (red lines) that are mapped to the regions. Locations of the stop-codon-containing exon 5, ARE and a poly-A signal sequence (pA) are marked. (B) Corresponding mouse Prox1 genomic sequence was also aligned against mouse EST database and the mapped ESTs are shown in read. Locations of the exon 5, ARE and a poly-A signal sequence (pA) are marked. (C) Cross-species sequence conservation analyses of the Prox1 3′-UTR from diverse primates, mammalian and vertebrates by using the University of California Santa Cruz (UCSC) Genome Brower revealed a high DNA sequence homology among various Prox1 3′-UTR. Thick black bar represents the conserved 3′-UTR of human PROX1. (D) Northern blot analyses showing human LEC PROX1 with an extended 3′-UTR. While lanes 1 and 2 were hybridized with a PROX1 open reading frame (ORF) probe, lanes 3, 4 and 5 respectively with the 3′-UTR probes P1, P2 and P3, of which locations are shown in panel A. (E) A 3′-RACE analysis for total RNA from primary human LECs demonstrates that human PROX1 mRNA terminates at 5,414-bp downstream from its stop codon. A putative poly-A signal sequence is marked with an asterisk. (F) Three highly conserved copies of the canonical AUUUA pentamer flanked by additional W (A or U) are present in the 3′-UTRs of dog, mouse, human, chicken and zebrafish Prox1 mRNAs. Asterisks mark the conserved bases. (G) A schematic diagram of the pTet-BBB vector constructs containing PROX1-ARE or c-fos-ARE in its 3′-UTR. Tet-promo, tetracycline-inducible promoter. (H) Northern blot analyses showing time-dependent decay of the β-globin mRNA containing no ARE (a negative control), PROX1-ARE or c-fos-ARE (a positive control) after tetracycline (doxycycline)-mediated shutdown of the transcription. β-globin DNA fragment was used as the probe and GAPDH mRNA was also shown as an internal loading control. (I) Intensity of the β-globin mRNA bands in panel H was quantified and normalized against GAPDH and the relative intensity was charted to show the stability of the β-globin mRNA with or without PROX1-ARE or c-fos-ARE.

We then set out to investigate the molecular mechanism underlying kaposin-B-induced PROX1 upregulation. Kaposin-B has been demonstrated to upregulate various cytokine genes by stabilizing their mRNAs through AREs located in their 3′-UTRs [57]. We thus examined the mRNA structure of the PROX1 gene. Although the open reading frame (ORF) of human or mouse PROX1 gene is about 2.2 kb long and encodes a 737-amino acid-long protein, reported northern blot analyses revealed that PROX1 transcript was as large as 8-kb in various tissues [60], [61], [62], suggesting that the PROX1 transcript has a long UTR at the 5′- and/or 3′ ends. In fact, we found that an 8-kb PROX1 transcript harboring an extended 3′-UTR has been annotated in a public genome database (ENST00000366958). However, the corresponding Prox1 transcript from mouse has not been annotated in the same public database. To further confirm the presence of PROX1 transcript with an extended 3′-UTR in both human and mouse, we aligned 7-kb downstream genomic sequences of human or mouse PROX1 against human or mouse expressed sequence tag (EST) databases and found that numerous EST sequences were mapped to the downstream of both human PROX1 and mouse Prox1 genes (Figure 3A&B), indicating that this region is indeed transcribed as a part of the fifth exon of PROX1 mRNA in both species. We then investigated sequence conservation of this 3′-UTR among Prox1 genes of other species. Analyses using a genome browser revealed a high DNA sequence homology in this extended 3′-UTR, especially in the second half, of the Prox1 genes from primates, placental mammals or vertebrates covering 48-speices [63] (Figure 3C).

Since PROX1 was expressed in multiple organs such as the brain, liver, muscle and heart [60], [61], [62] and most of EST fragments were derived from cDNA libraries of these organs, we asked whether LECs express PROX1 mRNA with a long 3′-UTR and thus performed northern blot analyses by using three different 3′-UTR probes and a PROX1 open reading frame (ORF) probe against RNAs isolated from human LECs. Indeed, both the 3′-UTR and the ORF probes detected a single ∼8-kb band (Figure 3D), indicating that LECs express a ∼8-kb long PROX1 mRNA. In addition, we performed the 3′-rapid amplification of cDNA end (RACE) analyses and found that a majority of human LEC-PROX1 mRNA terminates at 5,414-bp downstream from the termination codon of PROX1 (Figure 3E). We identified a classical poly-A signal sequence (AATAAA) at ∼20-bp upstream of the termination site. Together, our data demonstrate that PROX1 mRNA expressed in primary human LECs harbors a 5.4-kb-long 3′-UTR and that this unusually long 3′-UTR is conserved among the Prox1 genes of many vertebrate species.

### A functional AU-rich element is present in the PROX1 3′-UTR and promotes PROX1 mRNA turnover

Because kaposin-B stabilizes cytokine mRNAs through AREs in their 3′-UTR [57], we asked whether PROX1 mRNA contains a canonical ARE in its unusually long 3′-UTR. We performed bioinformatic analyses against the conserved vertebrate Prox1 3′-UTRs and found an AU-rich region approximately 400-bp upstream of the mRNA termination site (Figure 3F). Notably, this region contains three copies of the AUUUA core pentamer (its location marked in Figure 3A) that can be classified as a class I ARE [43], [44], [45], [46]. Moreover, these three tandem copies of core pentamer were also found to be conserved in Prox1 mRNA of dog, mouse, human, chicken and zebrafish (Figure 3F).

We next investigated whether the newly discovered PROX1-ARE can serve as an mRNA instability determinant by utilizing the classical β-globin mRNA stability reporter system (pTet-BBB) [64], [65]. In this system, the tetracycline (Tet)-controlled promoter directs the inducible expression of the rabbit β-globin gene and a specific DNA sequence element such as a putative ARE can be cloned into the 3′-UTR of the β-globin gene in order to evaluate its ability to destabilize otherwise stable β-globin mRNA [64], [65]. We cloned a 40-bp fragment containing the PROX1 AUUUA core pentamer in pTet-BBB (Figure 3G) and evaluated its effect on the stability of β-globin mRNA in NIH3T3/Tet-Off cells by northern blotting analyses. Indeed, the three AUUUA motifs from the PROX1 3′-UTR significantly destabilized the stable β-globin mRNA as potently as the c-fos ARE that was used as a positive control (Figure 3H), demonstrating that the 40-bp PROX1-ARE is sufficient to function as an instability determinant for PROX1 mRNA. Quantification of the northern blot bands revealed that while the unmodified β-globin mRNA shows a long half-life ( >210-minutes), the PROX1-ARE, like the c-fos-ARE, shortened the half-life of β-globin mRNA to ∼60-minutes (Figure 3I). Together, our data demonstrate that PROX1 mRNA contains an ARE functioning as an mRNA instability determinant in its unusually long 3′-UTR and that this newly identified PROX1-ARE may play an important role in the post-transcriptional regulation of PROX1 expression.

### HuR physically binds to PROX1-ARE and upregulates PROX1 expression

**Figure 4:**
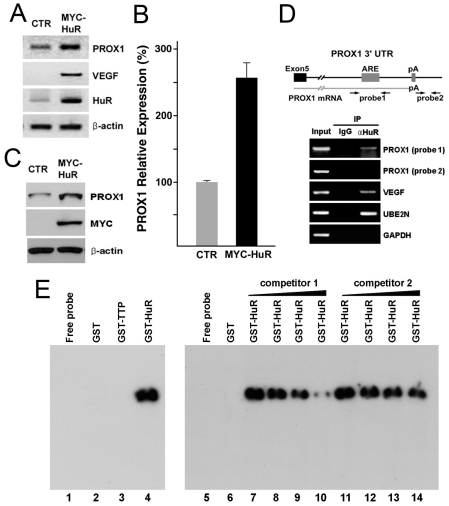
HuR upregulates PROX1-expression through physical interaction with the 3′-UTR of PROX1 mRNA. (A) A control (CTR) or a HuR-expression vector (MYC-HuR) was transfected into primary human LECs for 16 hours and the steady-state level of PROX1, VEGF, HuR and β-actin mRNAs was measured by semi-quantitative RT-PCR analyses. (B, C) In a separate experiment, a control (CTR) or a HuR-expression vector (MYC-HuR) was transfected into LECs for 16 hours and the expression of PROX1 was determined by qRT-PCR (B) and western blot (C) analyses. Expression of β-actin was used as the internal control for both assays. (D) Endogenous HuR protein forms a stable complex with PROX1 mRNA in LECs. HuR protein-PROX1 mRNA complex was immunoprecipitated from LEC-whole cell lysate (input) by a normal IgG (IgG) or anti-HuR antibody (αHuR) and resulting precipitates were subjected to RT-PCR analyses for PROX1, VEGF, UBE2N and GAPDH. Two neighboring primer pairs were used for PROX1 as shown in the upper panel: Probe-1 primer pair detects PROX1-ARE region (product size, 480-bp) and Probe-2, located at 25-bp downstream of PROX1 transcription termination site, serves as a negative control. VEGF and UBE2N mRNA are positive controls [49], [68] and GAPDH is a negative control for HuR-binding. (E) RNA-EMSA showing a complex formation between PROX1-ARE RNA and recombinant HuR protein. A PROX1-ARE RNA probe was *in vitro* transcribed and incubated with buffer alone (lane1), GST (lane2), GST-TTP (lane3), or GST-HuR (lane4) recombinant proteins and the RNA-protein complex formation was detected by polyacrylamide gel electrophoresis. Formation of PROX1-ARE RNA/HuR protein complex was inhibited by an increasing amount of unlabeled PROX1-ARE RNA probe (competitor 1) (lanes 5∼10), but not by unlabeled non-specific yeast total RNA (competitor 2) (lanes 11∼14).

We next investigated the molecular mechanism underlying the regulation of PROX1 mRNA stability through its ARE and searched for ARE-binding factors that may interact with the PROX1-ARE. The nuclear protein HuR, also known as ELAVL1, has been shown to be one of the best characterized ARE-binding proteins that bind various cytokine/chemokine mRNAs to increase their stability [66]. Therefore, we evaluated the possibility of HuR binding to the PROX1-ARE and promoting the mRNA stability. Toward this aim, a HuR-expressing vector was transfected into primary LECs and PROX1 expression was determined. Indeed, our semi-quantitative RT-PCR analyses showed that HuR overexpression resulted in upregulation of PROX1 as well as a known HuR-target gene VEGF [49], [67] (Figure 4A). This HuR-mediated PROX1 upregulation was also confirmed by using quantitative qRT-PCR (Figure 4B) and western blot (Figure 4C) analyses.

We next asked whether HuR protein can physically interact with the AU-rich region of the PROX1 3′-UTR and performed co-immunoprecipitation (co-IP) for a protein-RNA complex of endogenous HuR protein and PROX1 mRNA from primary LECs by using an anti-HuR antibody as previously described [68]. Precipitated protein-RNA complex was de-crosslinked, reverse-transcribed and PCR-amplified by using two neighboring sets of PROX1 PCR primers; Probe-1 and Probe-2. While Probe-1 primer pair detects the PROX1-ARE region, Probe-2 primer pair, 42-bp away from Probe-1, binds at 25-bp downstream from the end of PROX1 mRNA (Figure 4D). Importantly, whereas RT-PCR using Probe-1 amplified a corresponding product, RT-PCR using Probe-2 did not yield any product, indicating that the endogenous HuR protein is physically associated with the PROX1-ARE region and also that the Probe-1 product was not due to possible genomic DNA contamination in our co-immunoprecipitation assays (Figure 4D). As controls, we could detect the association of HuR protein with VEGF and UBE2N mRNAs [49], [68], but not with GAPDH mRNA (Figure 4D).

We next performed a RNA electrophoresis mobility-shift assay (RNA EMSA) to corroborate the molecular interaction between HuR protein and PROX1-ARE mRNA. We *in vitro* transcribed a RNA EMSA probe spanning the PROX1-ARE region and then investigated if this RNA probe can make a stable RNA-protein complex with recombinant HuR protein and/or another known ARE-binding protein tristetraprolin (TTP) [47]. Indeed, while GST alone or GST-TTP protein did not make any detectable RNA-protein complex, GST-HuR recombinant protein formed a stable complex with the PROX1-ARE RNA probe (Figure 4 E, lanes 1–4). It is interesting to find that HuR, but not TTP, binds to PROX1-ARE RNA probe, although both are known to have an affinity to the ARE motif [69]. Moreover, the complex formation between PROX1-ARE RNA probe and GST-HuR protein could be inhibited by an unlabeled specific competitor (PROX1-ARE RNA probe), but not by an unlabeled non-specific competitor (yeast total RNA), indicating a specific molecular interaction between HuR protein and PROX1-ARE region (Figure 4E, lanes 5∼14). Taken together, our data demonstrate that HuR protein physically interacts with PROX1 mRNA through the AU-rich region.

### Kaposin-B promotes PROX1 mRNA stability through HuR

**Figure 5:**
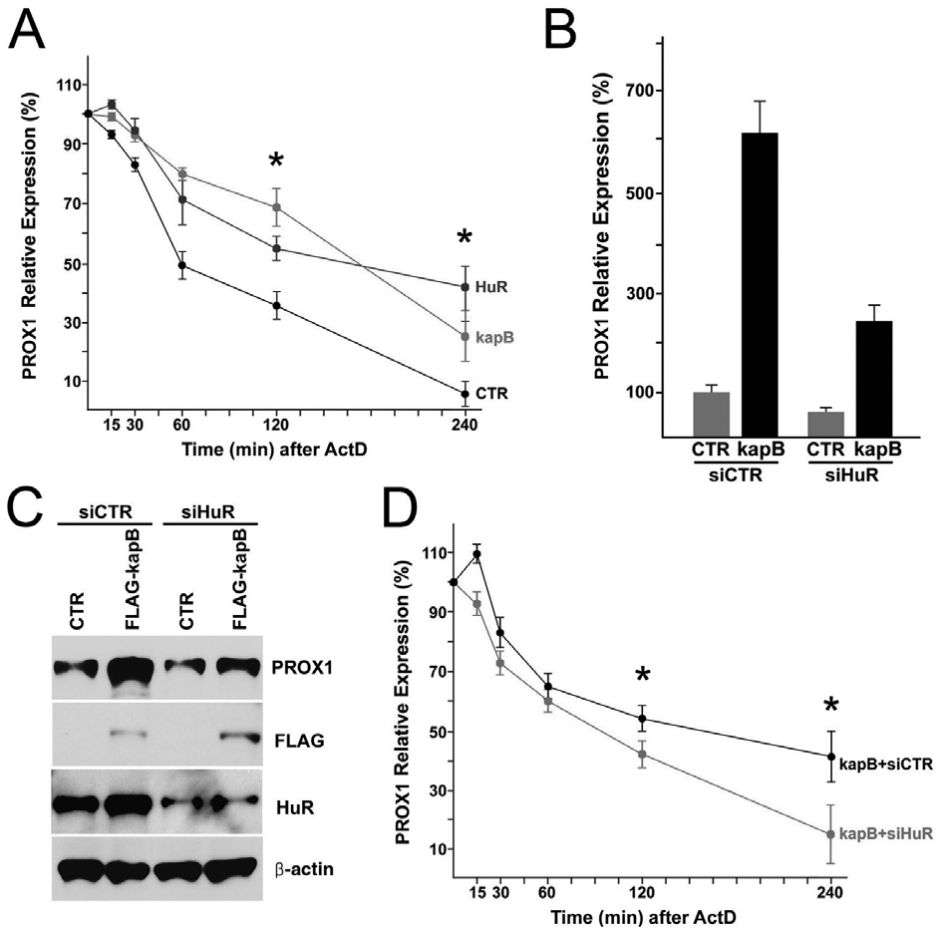
Kaposin-B upregulates PROX1 by promoting its mRNA stability through HuR. (A) PROX1 mRNA stability is increased by HuR and kaposin-B. LECs were transfected with a control (CTR), a HuR-expressing (HuR) or a kaposin B-expressing (kapB) vector for 16-hours and then treated with Actinomycin D (ActD) (2 µg/ml) for the indicated length of time. Total RNA was isolated and analyzed for PROX1 mRNA level by qRT-PCR analyses. (B,C) HuR is required for the kaposin-B-mediated PROX1 upregulation in LECs. LECs were transfected with a control (CTR) or a FLAG-tagged kaposin B-expressing (kapB) vector. After 16 hours, the control or kaposin-B-expressing cells were divided into two groups and then transfected again with siRNA against luciferase (siCTR) or HuR (siHuR). Total RNA and whole cell lysate was harvested from each group after 16-hours and subjected to qRT-PCR (B) or western (C) analyses. (D) Kaposin-B-mediated increase of PROX1 stability was abrogated by inhibition of HuR expression. LECs overexpressing kaposin-B were transfected with luciferase siRNA (kapB+siCTR) or HuR siRNA (kapB+siHuR) for 16-hours and then treated with Actinomycin D (ActD) (2 µg/ml). Total RNA was isolated at the indicated time points and analyzed for PROX1 mRNA level by qRT-PCR analyses. Similar results were obtained from three independent experiments and the error bars present standard deviations (SD) in a representative experiment. Asterisks in panels A &D present p-value less than 0.05.

Our findings of kaposin-B-induced PROX1 upregulation and HuR-binding to PROX1-ARE directed us to ask whether HuR and/or kaposin-B upregulate PROX1 by enhancing PROX1 mRNA stability. Toward this question, we overexpressed HuR or kaposin-B in primary LECs and quantified the steady-state level of PROX1 mRNA by qRT-PCR. Indeed, the ectopic expression of HuR or kaposin-B delayed the turnover of PROX1 mRNA in LECs and increased the half-life of PROX1 mRNA from ∼60 minutes in the control LECs to ∼180 minutes in LECs overexpressing HuR or kaposin-B (Figure 5A). We then asked whether HuR is required for kaposin-B-mediated PROX1 upregulation by knockdown of HuR in kaposin-B-expressing LECs. We found that HuR-knockdown significantly inhibited kaposin-B-mediated upregulation of PROX1 mRNA and protein determined by qRT-PCR and western analyses, respectively (Figure 5B&C). Moreover, we confirmed that this reduction in kaposin-B-mediated PROX1 upregulation is due to a decrease in PROX1 mRNA stability upon knockdown of HuR (Figure 5D). Together, our data demonstrate that kaposin-B upregulates PROX1 by promoting its mRNA stability through HuR.

### Kaposin-B stimulates cytoplasmic localization of HuR protein

**Figure 6:**
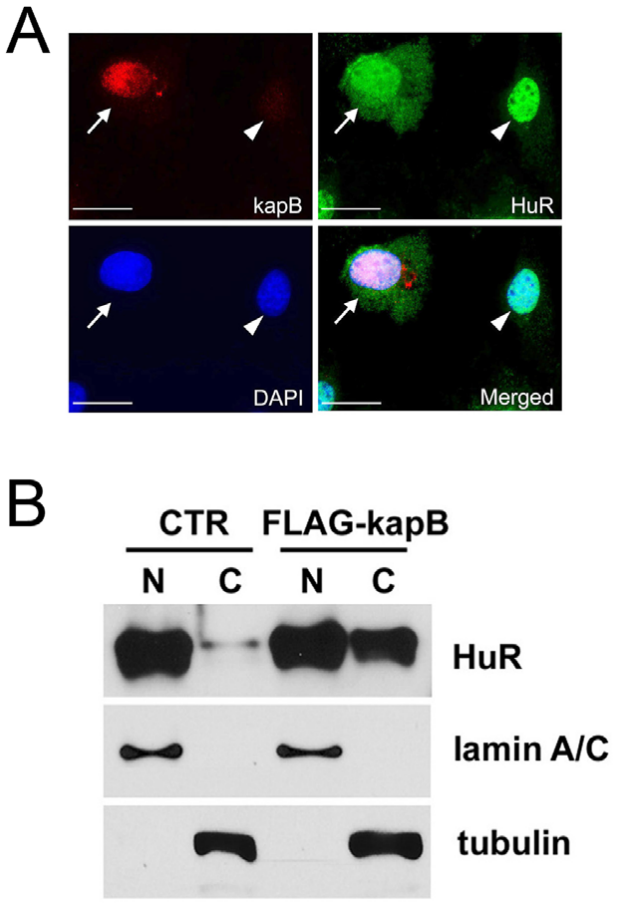
Cytoplasmic accumulation of HuR protein by kaposin-B. (A) LECs were transfected with an expression vector for FLAG-tagged kaposin-B for 16-hours and subjected to immunofluorescent analyses for FLAG-kaposin B (red), HuR (green) and DAPI (blue). A merged image shows that kaposin-B induces cytoplasmic accumulation of HuR only in kaposin-B-expressing cell (arrows), but not in a neighboring untransfected cell (arrowhead). Bar, 20 µm. (B) A control (CTR) or a FLAG-tagged kaposin-B vector (FLAG-kapB) was transfected into LECs for 16 hours. Nuclear (N) or cytoplasmic (C) fractions were collected and subjected to western blot analyses with antibodies against HuR, lamin A/C (nuclear marker) and tubulin (cytoplasm marker). Note accumulation of HuR protein in the cytoplasmic fraction by kaposin-B.

While HuR protein mainly resides in the nucleus, various cell stress signals activate cytoplasmic accumulation of HuR [52]. We next asked if kaposin-B activates localization of HuR protein to the cytoplasm where mRNA stability is regulated. Indeed, our immunofluorescent analyses revealed that ectopic upregulation of kaposin-B stimulated cytoplasmic mobilization of HuR protein (Figure 6A). Moreover, we harvested the cytoplasmic and nuclear fractions from control vs. kaposin-B-overexpressing LECs to quantify the amount of mobilized HuR by kaposin-B. Consistent with the immunostaining data, a significant amount of HuR protein was found to be exported to the cytoplasm (Figure 6B). Therefore, our data demonstrate that cytoplasmic accumulation of HuR protein is activated by kaposin-B, which may play an important role in PROX1 upregulation.

### The p38/MK2 kinase pathway is required for cytoplasmic accumulation of HuR protein and PROX1 mRNA stabilization

**Figure 7:**
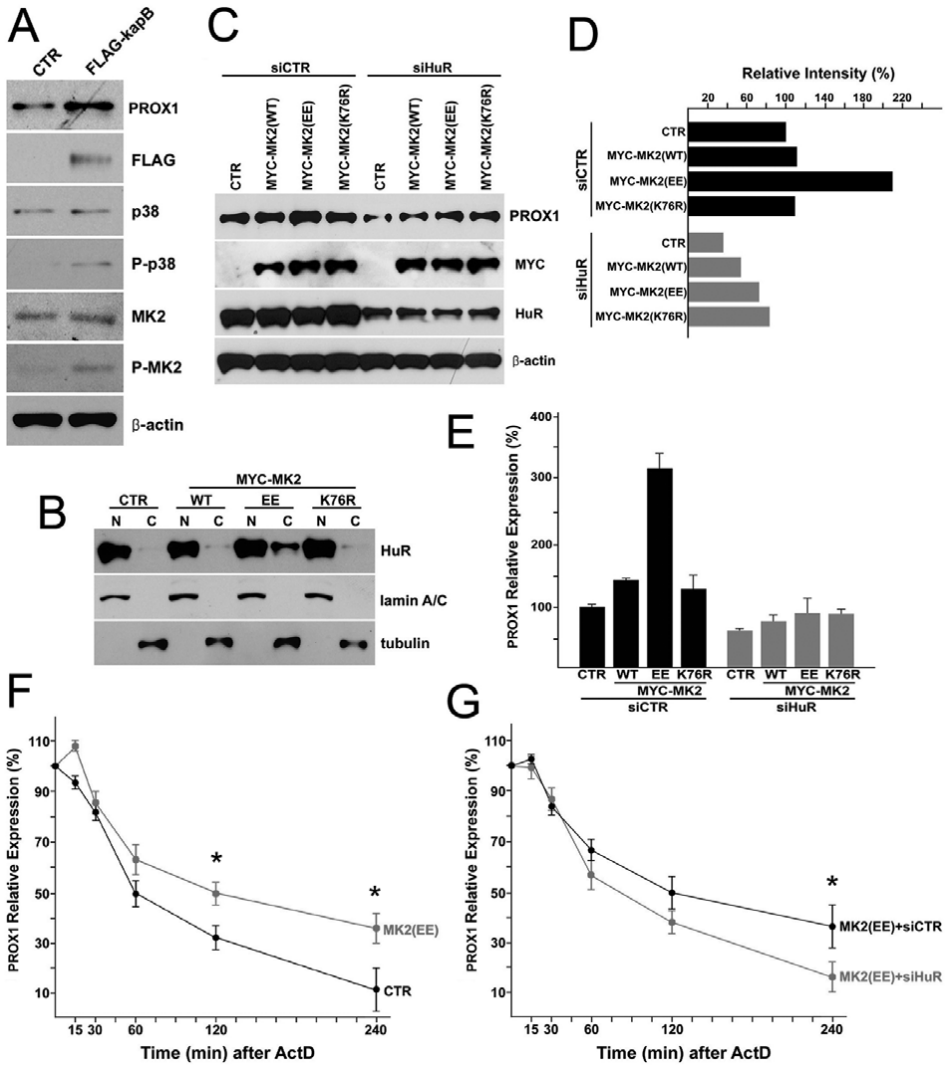
MK2 stimulated by kaposin-B activates the cytoplasmic accumulation of HuR protein and upregulates PROX1 mRNA stability. (A) Activation of p38 and MK2 by kaposin-B in LECs. LECs were transfected with a control (CTR) or a FLAG-tagged kaposin B-expressing vector (FLAG-kapB) for 16-hours and protein level of PROX1, FLAG-kaposin-B (FLAG), p38, phospho-p38 (P-p38), MK2, phospho-MK2 (P-MK2) and β-actin was determined by western blot analyses. (B) MK2 activation results in cytoplasmic accumulation of HuR protein. A control (CTR), a wild type-MK2 (WT), a constitutively active-MK2 (EE), or a dominant negative-MK2 (K76R) vector was transfected into LECs for 16 hours and the nuclear (N) and cytoplasmic (C) fractions were isolated and subjected to western blot analyses against HuR, lamin A/C (nuclear marker) and tubulin (cytoplasm marker). (C) HuR is required for MK2-mediated PROX1 upregulation. Expression of PROX1 protein was determined by western blot analyses in LECs that were transfected with a control (CTR), a wild type-MK2 (WT), a constitutively active-MK2 (EE) or a dominant negative-MK2 (K76R) vector. After 16 hours, transfected cells were divided into two groups and then transfected again with either luciferase siRNA (siCTR) or HuR siRNA (siHuR). Protein level of PROX1, Myc-tag, HuR and β-actin was determined after 16 hours. (D) Relative intensity of PROX1 bands in panel C was measured and charted in a graph. (E) MK2-mediated PROX1 upregulation is due to an increased expression of PROX1 mRNA. The steady-state level of PROX1 mRNA from the same set of experiment as panel (C) was determined by qRT-PCR. (F) MK2 activation increases Prox1 mRNA stability. A control (CTR) or a constitutive-MK2 (MK2 (EE)) vector was transfected into LECs for 16 hours and the steady-state level of PROX1 mRNA was determined by qRT-PCR analyses at the indicated time point (minutes) after Actinomycin-D (ActD) (2 µg/ml) treatment. (G) HuR is necessary for MK2-induced PROX1 stability. Constitutively active MK2 (EE) was overexpressed in LECs for 16 hours and the cells were then transfected again with luciferase siRNA (MK2 (EE)+siCTR) or HuR siRNA (MK2 (EE)+siHuR) for 16-hours, followed by Actinomycin-D (ActD) administration (2 µg/ml). PROX1 mRNA level at the indicated time points was determined by qRT-PCR analyses. Data are represented by mean and standard deviation (SD) and three independent experiments were performed to yield similar results. Asterisks in panels F and G present p-value less than 0.05.

Kaposin-B has been shown to activate the p38/MK2 pathway and stabilize various cytokine mRNAs [57], [58]. We further examined this previous observation in LECs and found that the ectopic expression of kaposin-B activated phosphorylation of p38 and MK2 proteins (Figure 7A). We then investigated whether activation of the p38/MK2 pathway is required for kaposin-B-mediated PROX1 upregulation. Notably, previous studies have shown that p38 MAPK promotes cytoplasmic accumulation of HuR in different cell types [70], [71], [72]. Therefore, we asked if activated MK2 can promote cytoplasmic localization of HuR protein in LECs and transfected LECs with vectors expressing a wild type, constitutively active (EE) or dominant negative (K76R) form of MK2 protein [73]. We found that whereas the constitutively active (EE) MK2 protein stimulated cytoplasmic accumulation of HuR, wild type or dominant negative MK2 protein did not (Figure 7B). Moreover, the expression of PROX1 was upregulated by constitutively active (EE) MK2 protein, but not by wild type or dominant negative MK2 protein, in LECs determined by western analyses (Figure 7C,D). Importantly, this upregulation of PROX1 by MK2 (EE) protein was abrogated by siRNA-mediated knockdown of HuR (Figure 7C,D), indicating that HuR is required for the kaposin-B/p38/MK2 pathway-mediated PROX1 upregulation. We also confirmed these findings by using quantitative qRT-PCR measuring PROX1 mRNA level (Figure 7E). Interestingly, we found that HuR knockdown slightly decreases PROX1 expression compared to control siRNA (Figure 7D,E). We think that this is because HuR protein is present at a basal level in the cytoplasm of LECs (Figure 6B) and may stabilize PROX1 mRNA under the normal condition and thus knockdown of HuR resulted in decrease of PROX1 expression. This speculation is supported by our endogenous HuR co-immunoprecipitation data (Figure 4D) demonstrating a stable complex formation between HuR protein and PROX1 mRNA in normal primary LECs.

We next asked whether MK2 (EE) protein-mediated PROX1 upregulation is due to PROX1 mRNA stabilization and thus studied the regulation of the PROX1 mRNA half-life by MK2 (EE) protein. Indeed, MK2 (EE) promoted PROX1 mRNA stability by increasing mRNA half-life by more than 60 minutes in LECs (Figure 7F). Moreover, we found that this increase in PROX1 stability by MK2 (EE) could be abrogated by knockdown of HuR (Figure 7G). Taken together, our data demonstrate that activation of the p38/MK2 pathway results in cytoplasmic accumulation of HuR protein and PROX1 upregulation through stabilization of PROX1 mRNA.

### The role of HuR in KSHV-mediated PROX1 upregulation

**Figure 8:**
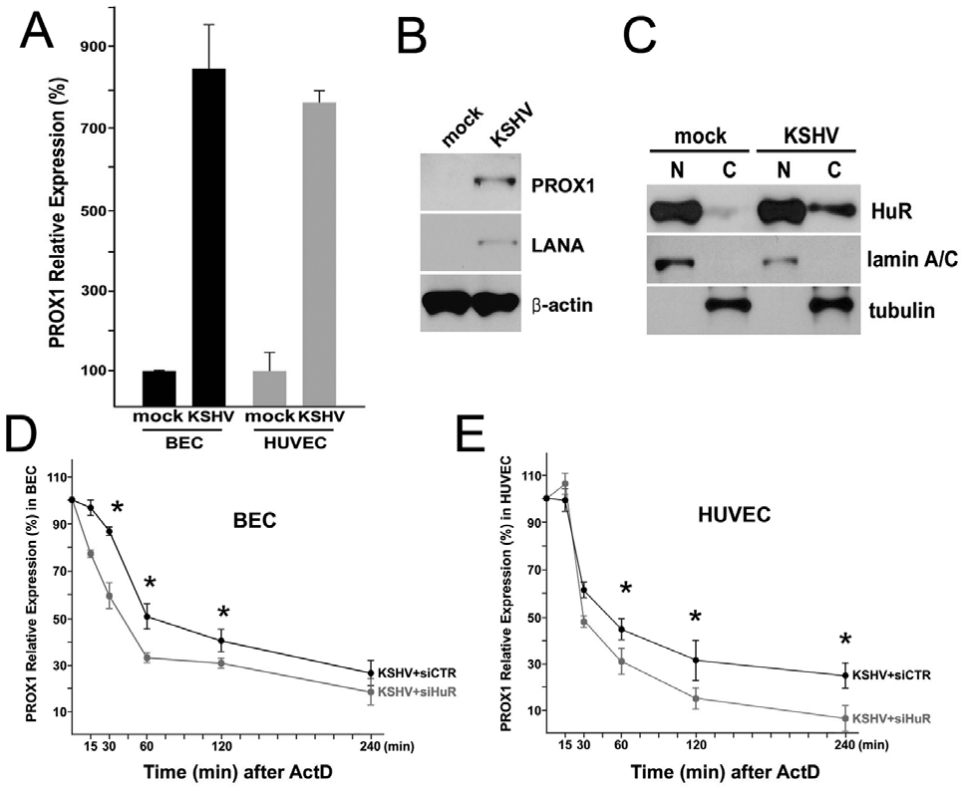
KSHV upregulates PROX1 by promoting its mRNA stability by HuR protein in primary BECs and HUVECs. (A) PROX1 is upregulated in KSHV-infected BECs and HUVECs. Primary BECs and HUVECs were infected with KSHV for 5 days and PROX1 expression was determined by qRT-PCR. (B) Protein level of PROX1 and LANA was determined in mock vs. KSHV-infected HUVECs. (C) KSHV-infection stimulates cytoplasmic accumulation of HuR protein in HUVECs. After KSHV infection for 5 days, cytoplasmic accumulation of HuR protein was assessed in the nuclear (N) and cytoplasmic (C) fractions. (D,E) HuR promotes PROX1 mRNA stabilization in KSHV-infected primary BECs and HUVECs. KSHV-infected primary BECs (D) and HUVECs (E) were transfected with luciferase siRNA (KSHV+siCTR) or HuR siRNA (KSHV+siHuR) for 16-hours and then treated with Actinomycin-D (ActD) (2 µg/ml). PROX1 mRNA level was determined at the indicated time points by qRT-PCR analyses. Three independent experiments were performed and data are expressed by mean and standard deviation (SD). Asterisks in panels D and E present p-value less than 0.05.

Our data above demonstrate the essential contribution of HuR in PROX1 upregulation by kaposin-B. We next asked whether HuR plays an important role in KSHV-mediated PROX1 upregulation in primary human BECs and HUVECs. We first confirmed the KSHV-mediated PROX1 mRNA upregulation in BECs and HUVECs (Figure 8A) and also PROX1 protein expression by KSHV in HUVECs (Figure 8B). We next investigated the cytoplasmic accumulation of HuR protein in KSHV-infected cells and found that KSHV infection resulted in a significant cytoplasmic localization of HuR protein in HUVECs (Figure 8C). Consistently, we also observed HuR cytoplasmic localization in KSHV-infected BECs (data now shown). We then asked whether HuR plays a role in the KSHV-mediated PROX1 upregulation by knockdown of HuR by siRNA in KSHV-infected BECs and HUVECs. Importantly, knockdown of HuR significantly decreased the half-life of PROX1 mRNA in KSHV-infected BECs and HUVECs (Figure 8D,E). In comparison, we were not able to measure the half-life of PROX1 mRNA in uninfected BECs or HUVECs due to their low/absent expression of PROX1. Taken together, our data demonstrate that KSHV infection stimulates cytoplasmic localization of HuR protein and that HuR plays an important role in KSHV-mediated PROX1 upregulation.

### Working model for KSHV/kaposin-induced PROX1 expression and lymphatic reprogramming

**Figure 9:**
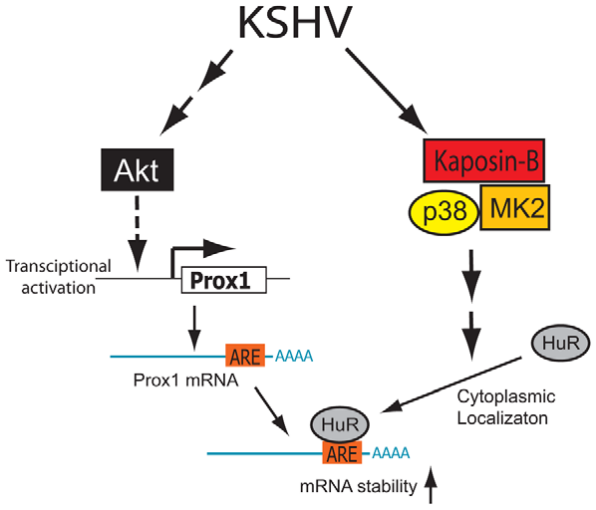
Proposed model of PROX1 mRNA stabilization and upregulation by KSHV and kaposin B. KSHV may employ two or more components for upregulating PROX1 in vascular endothelial cells: transcriptional activation and mRNA stabilization. While KSHV may stimulate the transcriptional activation of PROX1 through activation of Akt, as proposed by Morris et al [59], PROX1 mRNA may be stabilized by the KSHV latent gene kaposin-B, which activates the p38/MK2 pathway and cytoplasmic localization of HuR.

Based on our data presented here, we build a working model for the molecular mechanism underlying KSHV-mediated PROX1 upregulation (Figure 9). When KSHV infects vascular endothelial cells, the virus may employ two or more mechanisms for PROX1 upregulation: one may be a transcriptional activation of PROX1 possibly through Akt activation [59] and the other a post-transcriptional PROX1 mRNA stabilization by kaposin-B, which activates the p38/MK2 pathway. Activated MK2 by kaposin-B stimulates the nuclear export and cytoplasmic accumulation of HuR protein. Cytoplasmic HuR protein binds to the AU-rich region in the 3′-UTR of PROX1 mRNA and slows down PROX1 mRNA turnover, thus increasing the steady-state level of PROX1 mRNA in KSHV-infected cells.

## Discussion

KS tumor cells were reported to be derived from endothelium about 40 years ago [74]. However, the exact histogenetic origin of KS had remained uncertain because KS cells were found to express mixed cell-lineage markers of BECs and LECs [75]. Previously, we and others demonstrated that KSHV induces lymphatic reprogramming of vascular endothelial cells by upregulating the master control gene of lymphatic differentiation, PROX1 [20], [21], [22], [23]. This finding of endothelial cell fate reprogramming by KSHV has provided an important insight into the pathology of KS and KSHV. Nonetheless, the question how the virus induces the host cell fate change remained to be answered.

Our previous study revealed that the KSHV latent gene LANA only marginally induced expression of PROX1 (1.93-fold) [20]. Interestingly, recent two exciting studies have established a molecular connection between the function of LANA and PROX1 gene regulation: Di Bartolo et al. showed that KSHV LANA inhibits TGF-β signaling through epigenetic silencing of TGF- β type II receptor [76] and Oka et al. demonstrated that inhibition of TGF-β signaling upregulates PROX1 by ∼2 fold [77]. In spite of this intriguing molecular association, the degree of KSHV-induced PROX1 upregulation ( >8 fold) shown by us and others [20], [21], [22], [23] prompted us to hypothesize that LANA could not be the major activator for PROX1 upregulation in KSHV-infected cells and that another mechanism should also be present for KSHV-mediated PROX1 upregulation. This rationale directed us to search for an additional activator(s) among KSHV latent genes.

In this study, we investigated the role of kaposin-B, a latent gene of KSHV, in the KSHV-mediated PROX1 upregulation and found that kaposin-B promotes mRNA stability of PROX1. We defined the structure of PROX1 mRNA and identified a class I-type ARE in its 3′-UTR, through which PROX1 expression can be post-transcriptionally regulated by physiological or pathological stimuli. At this point, it seems that kaposin-B targets both class I (e.g., PROX1) and class II (e.g., GM-CSF [57]) mRNAs and the specificity determinant for kaposin-B targets needs to be further defined. Moreover, we discovered that the ARE-binding protein HuR is exported to the cytoplasm by kaposin-B and also by KSHV infection, and increase PROX1 mRNA stability. These findings are consistent with our observation from *in vitro* cell cultures and KS tumor samples that PROX1 upregulation occurs in only KSHV-infected cells, not in neighboring uninfected cells (Figure 1).

Kaposin-B-induced mRNA stabilization appears an attractive model for PROX1 upregulation since kaposin-B has been shown to upregulate other cytokine mRNAs such as GM-CSF and IL-6 [57]. However, it needs to be highlighted that kaposin-B is not the sole component in the molecular mechanism underlying PROX1-upregulation by KSHV because mRNA stabilization inevitably requires pre-existing mRNA and PROX1 mRNA is not expressed in BECs [24], [25]. Therefore, other factors/stimuli are needed for the initial transcriptional activation of the PROX1 mRNA synthesis. This hypothesis is further supported by two of our findings. First, PROX1-upregulation by kaposin-B was much prominent in LECs where PROX1 mRNA is already present, in comparison to BEC and HUVEC-backgrounds where PROX1 expression is fairly low, if any (Figure 2A). Second, PROX1 expression in BECs and HUVECs was more strongly activated by the entire virus (KSHV), compared to by kaposin-B alone (Figures 2A & 8A), again suggesting that kaposin-B alone is unable to efficiently activate PROX1 expression in BECs and HUVECs. Importantly, Morris et al has recently shown that Akt activation through gp130 is required for KSHV-mediated PROX1 upregulation and lymphatic reprogramming [59]. Considering the fact that KS tumors have been associated with numerous cytokines, chemokines and diffusible factors in their microenvironments [78], [79], [80], [81], it is highly likely that multiple KS-associated viral and/or cellular factors may activate the gp130/Akt pathway to prime the initial activation of PROX1 transcription [59]. We hypothesize that this transcriptional activation may require a subsequent secondary post-transcriptional mechanism involving kaposin-B to achieve PROX1 upregulation. This two-step mechanism is also consistent with the fact that PROX1-upregulation is limited to KSHV-infected cells. Taken together, Figure 9 illustrates our hypothesis that both steps (transcriptional activation and mRNA stabilization) may be necessary to achieve PROX1-upregulation and lymphatic reprogramming of blood vascular endothelial cells by KSHV.

On the other hand, kaposin is a unique KSHV latent gene considering distinct features in its transcription and translation. Kaposin transcripts are the most abundantly expressed viral mRNA throughout all stages of KS progression determined by in situ hybridization assays and a complex translational program directs production of multiple isoforms of kaposin gene product, termed kaposin A, B and C [82], [83]. Kaposin-B uses a non-conventional CUG start codon and consists of a series of tandem repeats of hydrophobic 23-amino acids, named DR1 and DR2 [82], [83]. DR2 can directly bind to MK2 and, when overexpressed, DR2 domain alone can dominantly inhibit the mRNA-stabilization function of the whole kaposin-B protein [57], [58]. It was also found that the DR1/DR2 repeats are more abundantly expressed in lytic- or TPA-treated cells [84] and that DR2 is reiterated three to five times in different stains of KSHV [58], suggesting a significant variation in the expression level and DR2 repeat number of kaposin-B. Accordingly, it will be interesting to investigate whether kaposin-B-induced PROX1 mRNA stabilization is more prominent during viral reactivation. Moreover, it is possible that both the expression level and structure of kaposin-B may affect its mRNA-stabilizing function through different ARE-binding proteins (including HuR) and target a different set of cellular mRNAs. It would be also exciting to study if kaposin-B may have other functions in addition to its role in mRNA stabilization.

Our current study brings up numerous questions. To date, the pathological role of PROX1 in KS development and progression has not been defined. Is the lymphatic phonotype more favorable for KSHV infection and propagation? Is lymphatic reprogramming a by-product or a goal of PROX1 upregulation by KSHV? Some insights may be obtained from interesting findings that PROX1 was shown to increase the invasion of endothelial tumor cells [85] and that PROX1 promotes the transition from benign to highly dysplastic phenotype in colon cancer [86]. While these studies support the oncogenic roles of PROX1, many other studies demonstrate the opposing role of PROX1 as a tumor suppressor [87], [88], [89], [90], [91], [92]. Therefore, cell type and tissue microenvironment may be crucial for PROX1 to play the oncogenic versus tumor suppressive role and further studies will be necessary to better understand the role of PROX1 in KS tumor development. Moreover, PROX1 has been reported to be important for cell-fate specifications in a broad range of cells including lymphatics [24], [25], liver [26], lens [27], [28], brain [29], [30], [31], [32], the ear [33], [34], [35], [36] and the heart [37], [38] during development and has been associated with post-developmental processes such as cell cycle regulation [28], [39], [93] and inflammation [94]. It will be very interesting to investigate whether PROX1 is post-transcriptionally regulated for any of its functions during and after development.

## Materials and Methods

### Cell cultures and transfection

Human primary dermal blood vascular endothelial cells (BECs) and lymphatic endothelial cells (LECs) were isolated from anonymous neonatal human foreskins and cultured as previously described [95] with an approval of the University of Southern California Internal Review Board (PI: YK Hong). Primary human umbilical venous endothelial cells (HUVECs) were purchased from Lonza (Basel, Switzerland), and cultured in EGM-2 medium (Lonza). NIH3T3 cells containing tTA (Tet-Off), named B2A2, were kindly provided by Dr. Ann-Bin Shyu (University of Texas Houston Health Science Center) [64], [65], [96]. NIH3T3 cells were transfected by using Lipofectamine 2000 (Invitrogen) and primary endothelial cells were transfected by electroporation (Nucleofactor II, Amaxa Biosystems).

### Plasmid constructs

The pTet-BBB and pTet-BBB-Fos-ARE vectors were kindly provided by Dr. Ann-Bin Shyu (University of Texas-Houston Medical School) [64], [65]. pTet-BBB contains the Tetracycline (Tet) - responsive element that drives transcription of the rabbit β-globin reporter gene and pTet-BBB-Fos-ARE bears the Fos-ARE inserted in the 3′ UTR of the reporter. To make pTet-BBB-Prox-ARE that contains the 40-bp PROX1-ARE (4,994 ∼5,034 bp downstream from the stop codon of human PROX1), sense and anti-sense primers harboring the ARE and BamHI-half site at the both ends (gatccTGCATAATTTATTGGTTTAATTTATCCTAATTTATTTGATG, gatccATCAAATAAATTAGGATAAATTAAACCAATAAATTATGCAG) were annealed and cloned at the unique BglII site of the 3′-UTR of pTet-BBB. To clone the human PROX1 3′-UTR, a 5.4-kb fragment covering the human PROX1 3′-UTR was amplified by two primers (ATTAGCGGCCGCTTTGAATGTATGAAGAGTAGCAGTCC, AATCAAACGGCACTGAGCTT) from a bacterial artificial chromosome (RPCI11-71F10, Invitrogen) and was cloned in pCRII-Blunt (Invitrogen). Expression vectors encoding MYC-tagged MK2 (WT, EE, and K76R) were kindly provided by Dr. Matthias Gaestel (Hannover Medical School, Germany) [73]. Expression vectors for myc-tagged HuR and FLAG-tagged kaposin-B were kind gifts by Drs. Dominique Morello (Institute Pasteur, France) [97] and Craig McCormick (Dalhousie University, Canada) [57], respectively. SiRNA for HuR was purchased from Santa Cruz Biotechnology (siHuR; cat. sc-35619) and the control siRNA (CUUACGCUGAGUACUUCGATT, UCGAAGUACUCAGCGUAAGTT) against the firefly luciferase was previously described [95].

### Rapid amplification of cDNA ends (RACE)

3′-RACE assay was performed by following the manufacturer's instruction (First Choice RLM Race kit, Applied Biosystems). Total RNA was isolated from human primary LECs and subjected to 3′ RACE by using two sets of PCR primers (GGATTGGTCTCAGCGCTACC, GCGAGCACAGAATTAATACGACT; AACTGAACTGATAAAGTCAATTTTTG, CGCGGATCCGAATTAATACGACTCACTATAGG). Amplified PCR products were cloned in the pGEM-T Easy vector (Promega) and sequenced to define the end of PROX1 mRNA.

### KS and KSHV production

De-identified anonymous KS specimens were obtained from the AIDS and Cancer Specimen Resource (ACSR) with an approval of the University of Southern California Internal Review Board (PI: YK Hong). KSHV was purified from BCBL-1 cells by following a previous description [23] with a minor modification. Briefly, BCBL-1 cells were cultured to the density of 5∼10 million cells/ml and then activated with TPA (20 ng/ml) and sodium butyrate (NaB, 3 mM). After 24∼48 hours, TPA/NaB-containing media was replaced with normal media and cells were incubated for additional 5 days. Culture media was then collected and filtered through 0.45-µm filters and centrifuged for 20 minutes at 4°C at 8,000 rpm to remove cell debris. Supernatant was centrifuged for 5 hours at 4°C at 11,000 rpm to pellet the virus, which was then resuspended in endothelial cell media. Infectivity was measured by immunohistochemistry for LANA after infection for 5 days.

### RNA EMSA and RNA/protein immunoprecipitation

GST-HuR and GST-TTP fusion proteins were isolated as previously described [98]. Expression vectors for GST-HuR and GST-TTP were kindly provided by Drs. Henry Furneaux (University of Connecticut) [51] and Gilles Pages (University of Nice-Sophia Antipolis, France) [99], respectively. For RNA EMSA experiments, radio-labeled RNA transcripts (200 Kcpm/reaction) was mixed with 200 ng GST fusion proteins or GST alone in a previously described binding buffer [100]. The reaction mixture was incubated for 30 minutes at 30°C and treated for 15 minutes at room temperature with 100 U of Ribonuclease T1 (Roche). For competition assays, specific (unlabeled PROX1-ARE RNA probe) or nonspecific (yeast total RNA) competitors were incubated for 15 minutes at 30°C with the proteins in the binding buffer before the addition of the labeled transcripts. The reaction mixtures were resolved on 8% native polyacrylamide gels in 0.5× Tris borate-EDTA (TBE) buffer. Gels were dried and exposed to X-ray film. RNA/protein immunoprecipitation assay was performed essentially as described [96] by using protein A/G-Sepharose beads pre-incubated with anti-HuR (3A2, SC-5261, Santa Cruz Biotechnology). RNA was isolated from supernatants and reverse-transcribed with Superscript II (Invitrogen). The transcripts were amplified by PCR by using the following primers: PROX1 (probe-1), ATCCTAATTTATTTGATGAAGGTG, TGCACATACATTCAGTTTAAGAGG; PROX1 (probe-2), TCAGTGCCGTTTGATTTTCTTAAA, GGAACA TCTTTCCTTGTTCTTAGA; VEGF, TCCAATCTCTCTCTCCCTGAT, CGGATAAACAGTAGCACCAAT [67]; and UBE2N, TACCCAATGGCAGCCCCTAA, TTCCACTGCTCCGCTACATCA [68]. The resulting PCR products were analyzed by 2% agarose gels.

### Immunostaining and western blot analyses

Cells were cultured on 8-mm cover slips and infected with KSHV for 5∼7 days or transfected with FLAG-tagged kaposin-B for 16 hours. Cells were then fixed with 4% paraformaldehyde for 10-minutes, washed in phosphate-buffered saline solution (PBS) and treated with blocking solution (5% Bovine serum albumin) overnight. Subsequent immunostaining was performed as previously described [95]. Antibodies and dilution factors used for immunofluorescent staining analyses are follows; PROX1 (1∶1000, ReliaTech, Germany), LANA (1∶1000, Advanced Biotechnologies Inc, Maryland), HuR (1∶1000, Santa Cruz Biotechnology), FLAG tag (1∶1000, Sigma-Aldrich). Antibodies used for western analyses were PROX1 (1∶1000, Millipore Corporation, MA), β-actin and FLAG tag (all in 1∶2000, Sigma-Aldrich Corporation), MYC tag (1∶1000, Covance), lamin A/C, p38 and phospho-p38 (all in 1∶1000, Cell Signaling Technology), tubulin, MK2, phospho-MK2, and HuR (all in 1∶1000, Santa Cruz Biotechnology).

### Northern blot analyses

Total RNA was purified from primary human LECs, separated in an agarose gel, transferred to nylon membrane and then hybridized with ^32^P-labeled DNA probes. A 2.2-kb fragment was prepared as the PROX1 ORF probe by digesting pcDNA3-hPROX1 with NotI [98]. Three 3′-UTR probes, P1 (∼2.3-kb), P2 (∼0.5-kb) and P3 (∼0.7-kb), were prepared by EcoRV, XbaI/XmnI, and AfeI/SpeI digestions from PROX1 5.4 kb 3′ UTR, respectively. For the functional test of PROX1-ARE, NIH3T3/Tet-Off cells were transfected with pTet-BBB, pTet-BBB-Prox-ARE or pTet-BBB-Fos-ARE [64], [65] and were grown in doxycycline (40 ng/ml)-containing DMEM/FBS medium for 48-hours. Transcription of β-globin was induced by removing doxycycline (replacing media with doxycycline-lacking media) for 110-minues. Subsequently, doxycycline (500 ng/ml) was added to the media to shut down the transcription and total RNA was harvested after 0, 30, 60, 90, 120, 180 and 240-minutes. Northern blot analysis was performed by using ^32^P-labeled β-globin and GAPDH probes.

### Quantitative real time and conventional RT-PCR

Real-time RT-PCR (qRT-PCR) was performed by using TaqMan EZ RT-PCR Core Reagent (Applied Biosystems). For dual-labeled probe-based qRT-PCR reactions, each reaction was multiplexed for both target gene and the internal control β-actin for normalization. Conventional RT-PCR was performed by using Superscript II (Invitrogen) and Taq polymerase (New England Biolabs). Primer sequences will be provided upon request.

## Supporting Information

Figure S1 Regulation of lymphatic-signature genes by kaposin-B in LECs, BECs and HUVECs. A control vector (CTR) or a kaposin B-expressing vector (kapB) was transfected into LECs, BECs and HUVECs for 16 hours and the expression level of podoplanin (A), VEGFR-3/flt4 (B), LYVE-1 (C), FGFR-3 (D), SLC (E) and p57 (F) was determined and normalized against the internal control β-actin by using quantitative real time RT-PCR (qRT-PCR) analyses.
(4.02 MB TIF)
