# Supplementary figures and images for: Kaposin-B Enhances the PROX1 mRNA Stability during Lymphatic Reprogramming of Vascular Endothelial Cells by Kaposi's Sarcoma Herpes Virus
Source: PLoS Pathog. 2010 Aug 12;6(8):e1001046. doi: 10.1371/journal.ppat.1001046 (PMC2921153; doi:10.1371/journal.ppat.1001046)

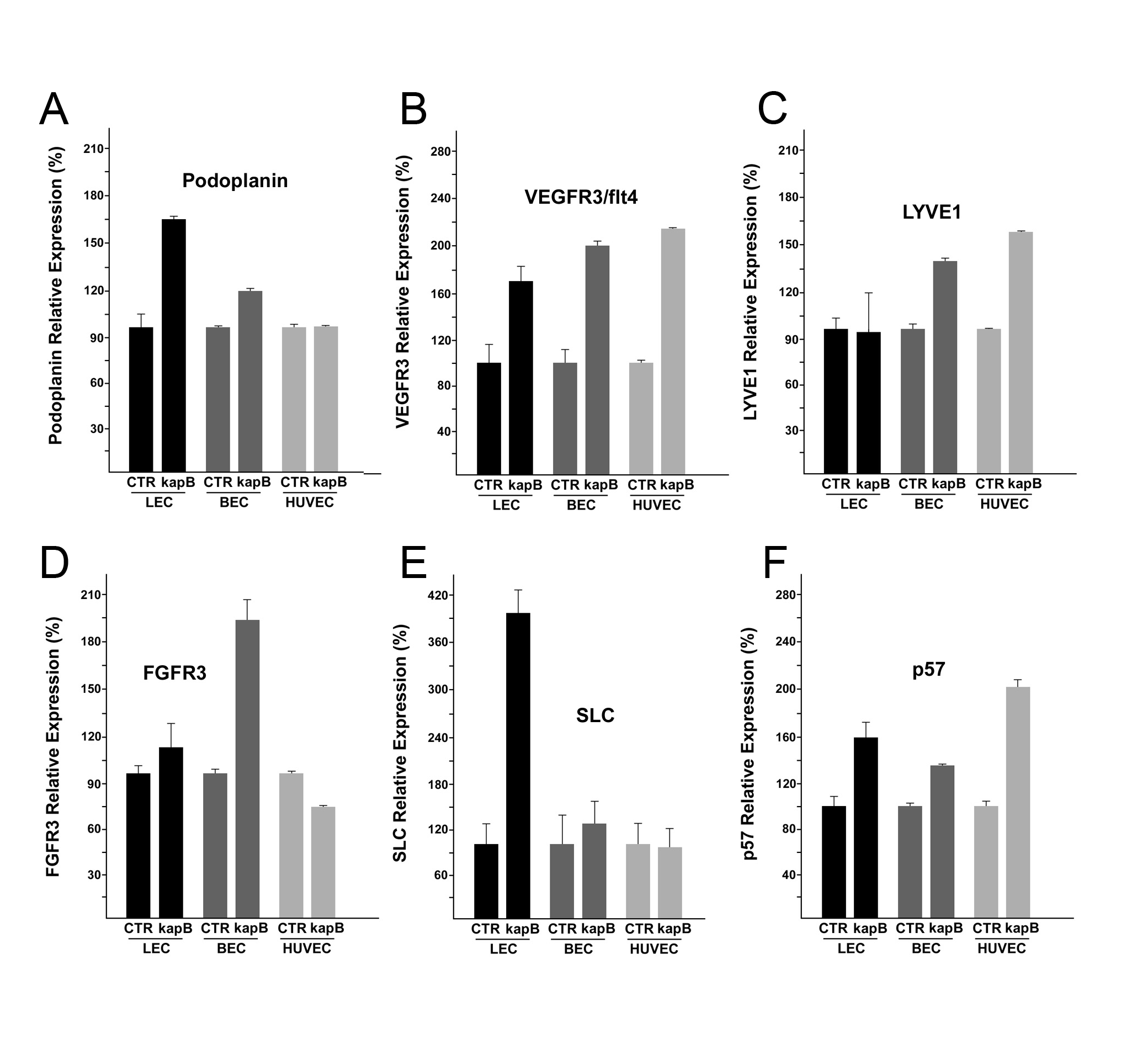

Supplement: Figure S1 — Regulation of lymphatic-signature genes by kaposin-B in LECs, BECs and HUVECs. A control vector (CTR) or a kaposin B-expressing vector (kapB) was transfected into LECs, BECs and HUVECs for 16 hours and the expression level of podoplanin (A), VEGFR-3/flt4 (B), LYVE-1 (C), FGFR-3 (D), SLC (E) and p57 (F) was determined and normalized against the internal control β-actin by using quantitative real time RT-PCR (qRT-PCR) analyses. (4.02 MB TIF) [file ppat.1001046.s001.tif]
